# Application of a 3D fusion model to evaluate the efficacy of clear aligners: A clinical study

**DOI:** 10.6026/973206300212124

**Published:** 2025-07-31

**Authors:** Zeeshan Mubashir, Ravi Ranjan, Swathi Jayan, Abida Parveen, Archana H., Dhananjay Rathod, Rahul Tiwari

**Affiliations:** 1 Department of Orthodontic and Dentofacial Orthopaedics, Al Jouf Specialised Dental Center, Ministry of Health, Sakaka, Aljouf Province Kingdom of Saudi Arabia; 2 Department of Orthodontics and Dentofacial Orthopedics, Deoghar, Jharkhand, India; 3 Department of Orthodontics and Dentofacial Orthopedics, HKDET's Dental College Humnabad, Karnataka, India; 4 Department of Orthodontics and Dentofacial Orthopaedics, MMCDSR, Maharishi Mahakandeshwar University, Mullana, Ambala, Shimla, Himachal Pradesh, India; 5 Department of Orthodontics and Dentofacial Orthopedic, HKDETS dental college and hospital humnabad, Bidar, Karnataka, India; 6 Department of Dentistry, Sheikh Bhikhari Medical College, Hazaribagh, Jharkhand, India; 7 MA, Political Science, Andhra University, Visakhapatnam, Andhra Pradesh, India

**Keywords:** Clear aligners, 3D fusion model, tooth movement accuracy, orthodontics, treatment prediction

## Abstract

Clear aligners have become a popular orthodontic treatment alternative due to their aesthetic and hygienic advantages. A prospective clinical research was conducted on 30 patients undergoing aligner therapy. Mesiodistal and vertical movements showed minimal discrepancy (mean <1 mm), with no significant differences. However, rotation, angulation and torque demonstrated significant deviations (p < 0.05), with over 40% of teeth showing severe discrepancies in these categories. A 3D fusion model enhances clinical accuracy by identifying movement-specific discrepancies, aiding in treatment planning and refinements.

## Background:

The pursuit of enhanced esthetics and patient comfort has led to the growing preference for clear aligners in contemporary orthodontics. Unlike conventional fixed appliances, aligners offer a removable, transparent and customized alternative for tooth movement, making them particularly appealing to adult patients. Their aesthetic appeal, improved hygiene maintenance and reduced risk of enamel decalcification have significantly contributed to their widespread clinical adoption [1, 2-3]. However, the challenge remains in objectively evaluating their effectiveness in achieving predicted tooth movements, particularly when treatment planning is carried out digitally using advanced software systems. The integration of "three-dimensional (3D)" imaging technology in orthodontics has revolutionized diagnostic and monitoring capabilities. Fusion models that combine intraoral scans, "cone-beam computed tomography (CBCT)", and digital treatment simulations provide a comprehensive platform to assess treatment outcomes in a quantifiable manner [4, 5-6]. These 3D fusion models allow clinicians to superimpose predicted and achieved tooth positions, facilitating a more precise assessment of aligner efficacy. This approach has become increasingly valuable in evaluating specific parameters such as rotation, tipping, intrusion and bodily movement, which are traditionally more challenging to achieve with clear aligners compared to fixed appliances [7]. Recent studies suggest that discrepancies often exist between virtual setups and clinical outcomes, largely due to biological variability, patient compliance, and limitations of aligner mechanics [8]. Evaluating these discrepancies using a 3D fusion model provides deeper insights into the performance of aligners and highlights the need for refinements or mid-course corrections [9]. This is especially important as aligner systems continue to evolve with newer materials, improved biomechanical designs and adjunctive aids like attachments and elastics. It further seeks to identify the types of tooth movements most prone to deviation, thereby guiding future improvements in aligner-based orthodontic protocols [10]. Therefore, it is of interest to apply a 3D fusion model to clinically assessing the accuracy of predicted versus actual tooth movements in patients undergoing aligner therapy.

## Materials and Methods:

This prospective clinical research was conducted on 30 patients undergoing orthodontic treatment with clear aligners at a university-based orthodontic clinic. The inclusion criteria comprised individuals aged 18-35 years with Class I malocclusion, mild to moderate crowding or spacing (<5 mm), and no history of previous orthodontic treatment. Patients with craniofacial anomalies, missing teeth (except third molars), or periodontal disease were excluded. Each participant underwent an "initial digital intraoral scan (T0)" using an iTero® scanner. A digital treatment plan was prepared using proprietary aligner software, generating a final predicted model (T1-virtual). Upon completion of the last aligner in the series (mean duration: 7 months), a second intraoral scan (T2) was recorded. The T1-virtual and T2 scans were imported into Geomagic® Control X software and fused with the baseline T0 scan to create a superimposed 3D fusion model for each case. Tooth movements were evaluated in six directions: mesiodistal, buccolingual, vertical (intrusion/extrusion), rotation, angulation, and torque. Linear and angular differences between predicted (T1) and achieved (T2) positions were measured using semi-automated tools. An average of three teeth per arch-central incisor, canine, and first premolar was selected for detailed evaluation. The degree of discrepancy was categorized as mild (<1 mm or <2°), moderate (1-2 mm or 2-5°), or severe (>2 mm or >5°). Descriptive statistics and paired t-tests were applied to assess significance. A p-value <0.05 was considered statistically significant.

## Results:

The 3D fusion model analysis revealed notable differences between the predicted and achieved tooth positions in several dimensions of movements. The most accurate movements were observed in mesiodistal translation and vertical changes (intrusion/extrusion), while the greatest discrepancies occurred in rotation and torque, especially in posterior teeth. The mean discrepancy between predicted and actual movements across six directions is shown. Mesiodistal and buccolingual movements demonstrated relatively minor differences (mean <1 mm), whereas rotation discrepancies exceeded 3° on average. Statistically significant differences were noted for rotation (p = 0.002), torque (p = 0.010), and angulation (p = 0.034), indicating that these movements are less predictably achieved by aligners (Table 1 - see PDF). The percentage of teeth falls into mild, moderate or severe discrepancy categories. Over 60% of mesiodistal and vertical movements were classified as mild. In contrast, more than 40% of rotational and torque discrepancies fell into the severe category (>5°), underlining the mechanical limitations of clear aligners in achieving complex movements (Table 2 - see PDF). These findings emphasize the relative precision of clear aligners in achieving linear tooth movements, while highlighting the need for adjunctive aids or refinements in cases requiring rotational or torque corrections.

## Discussion:

This research aimed to evaluate the efficacy of clear aligners by comparing predicted and achieved tooth movements using a 3D fusion model. The findings suggest that while aligners perform well in achieving linear tooth movements such as mesiodistal and vertical displacement, their accuracy significantly decreases for complex movements like rotation, torque and angulation. The minimal discrepancies in mesiodistal and vertical changes observed in this research align with previous evidence indicating that aligners are effective in translating teeth along simpler vectors, especially when attachments and interproximal reduction are properly utilized [11]. The predictability in vertical movement may also be attributed to the aligner's ability to control extrusion and intrusion within anterior teeth, though intrusion of molars remains challenging. In contrast, the significant deviations in rotational and torque movements underscore mechanical limitations. Rotation, especially of cylindrical teeth like canines and premolars, has long been considered one of the least predictable movements in aligner therapy [12]. This may be due to the aligner's flexible material not fully engaging the tooth's surface, leading to insufficient rotational force. Similarly, torque control requires a precise force system and root movement, which is not easily achievable with plastic aligners alone without biomechanical enhancements [13]. The high proportion of moderate to severe discrepancies in torque and rotation indicates a need for refinements during treatment or incorporation of auxiliary techniques such as attachments, optimized cutouts, or even hybrid treatment approaches. Recent studies support the use of overcorrections in the digital setup to account for such deviations, a method that could be considered in future protocols [14]. The 3D fusion model provided an objective, quantifiable tool for assessing treatment accuracy. By superimposing pre-treatment and post-treatment data, clinicians can now visualize and measure specific shortfalls in treatment, allowing for individualized refinements and better patient outcomes [15]. The use of a 3D fusion model has been shown to enhance the accuracy of clear aligner therapy evaluation and provide a reliable clinical reference for treatment planning [16]. However, limitations such as sample size and short follow-up duration must be acknowledged. Future studies with larger, multi centric samples and stratification based on tooth type or movement complexity could enhance understanding of aligner performance. Overall, this research emphasizes that while clear aligners are a valuable modality in orthodontics; their efficacy is movement-dependent. Clinicians should remain vigilant about their limitations and plan treatment accordingly [17, 18, 19-20].

## Conclusion:

Clear aligners are effective in achieving planned mesiodistal and vertical tooth movements with minimal discrepancy. However, their efficacy significantly declines when executing complex movements such as rotation, torque, and angulation, which often require mid-course corrections or auxiliary aids.
